# Impact of Hypocaloric Diets on Weight Loss and Body Composition in Obese Dogs: A Meta-Analysis

**DOI:** 10.3390/ani15020210

**Published:** 2025-01-14

**Authors:** Karoline Vanelli, Rafael Fernando Wisneski, Camila Estevão, Fernanda Caroline Mayer, Leandro Batista Costa, Saulo Henrique Webber, Cláudia Turra Pimpão

**Affiliations:** Department of Animal Science, Pontíficia Universidade Católica do Paraná, Curitiba 80215-901, Brazil; rafaelwisneski@gmail.com (R.F.W.); dracamilaestevao@gmail.com (C.E.); fernanda.c.mayer@animaeducacao.com.br (F.C.M.); batista.leandro@pucpr.br (L.B.C.); saulo.weber@pucpr.br (S.H.W.)

**Keywords:** canines, nutritional components, overweight, systematic review

## Abstract

Canine obesity is a complex, multifactorial condition marked by excessive body fat accumulation due to a sustained positive energy balance. Over the past decade, its global prevalence has risen significantly in most industrialized nations. Despite the availability of numerous commercial diets designed for obesity management in dogs, these products exhibit considerable variability in nutrient composition, and there is currently no standardized guideline on optimal macronutrient levels for effective weight loss. This study aimed to evaluate the effects of specific macronutrient levels on weight loss and body composition in obese dogs. A comprehensive literature search was conducted from 2022 to 2024 across PUBMED, Scielo, Web of Science, and Google Scholar. Out of an initial 1727 documents, 20 studies met the inclusion criteria and were incorporated into this meta-analysis. Diets with energy densities below 3.275 kcal, protein levels above 25%, total dietary fiber exceeding 12%, lower fat (<10%), and reduced non-nitrogenous extract (<40%) demonstrated beneficial effects on all evaluated parameters, including weight loss and body composition assessment. This meta-analysis provides evidence-based nutritional recommendations on optimal caloric, protein, fiber, fat, and carbohydrate levels for hypocaloric diet formulations, supporting healthy weight loss and lean mass preservation in obese dogs. These findings contribute to the development of effective dietary strategies that enhance canine quality of life and longevity.

## Simple Summary

Obesity is among the most prevalent nutritional disorders in dogs, marked by an excessive accumulation of body fat. Effective treatment typically involves the implementation of hypocaloric diets combined with increased physical activity. This systematic review and meta-analysis assessed the effects of various dietary components—namely protein, fat, fiber, and carbohydrates—along with overall energy intake, on the weight and body composition of obese dogs to determine optimal nutritional levels for promoting weight loss. After analyzing data from 20 studies, the results indicate that diets with elevated protein (>25%) and fiber (>12%) levels support weight reduction and help preserve lean muscle mass, whereas lower fat (<10%) and carbohydrate (<40%) levels are associated with reductions in body fat. Conversely, diets high in carbohydrates were linked to weight gain. Additionally, individual dog characteristics significantly influenced the outcomes. These findings offer valuable insights for optimizing hypocaloric diet formulations, facilitating an effective and health-promoting weight-loss process in canine patients.

## 1. Introduction

Obesity is a multifactorial nutritional disorder characterized by an excessive accumulation of body fat, generally defined as a body weight exceeding the ideal by 15–20% [1]. This condition has escalated to epidemic proportions in both developed and developing nations, affecting approximately 55% of dogs in the United States and 44% in Brazil [2,3]. Higher prevalence is observed among female dogs, adults or older animals (aged 5–10 years), and neutered individuals. Additionally, various environmental, social, and cultural factors—alongside caregiver attributes, such as dietary habits, lifestyle, and attachment to the animal—contribute to the complexity of obesity [3].

The primary contributors to canine obesity are high-calorie food consumption and insufficient physical activity. Excessive caloric intake combined with decreased energy expenditure promotes a positive energy balance, leading to fat accumulation and alterations in body composition [4,5,6].

The therapeutic approach for managing canine obesity involves enrolling the animal in a structured weight reduction program. This program requires caregiver awareness and cooperation, the implementation of specialized low-calorie diets, a physical activity plan, and regular monitoring of the animal’s progress [7,8]. Typically, the degree of caloric restriction is determined based on an estimate of the caloric intake needed to achieve the ideal weight, calculated in reference to the animal’s current body condition score [7].

The primary objectives of nutritional intervention are to facilitate effective and gradual weight loss, preserve lean muscle mass, promote satiety, increase energy expenditure, dilute dietary energy density, and regulate postprandial glycemic response [8,9].

Despite progress in canine obesity management, the ideal levels of caloric restriction intake and optimal nutrient concentrations for effective weight control remain subjects of debate. Research suggests that energy restriction intake can increase hunger, promote persistent food-seeking behavior, and exacerbate anxiety, which may lead caregivers to abandon weight reduction programs for their pets [10,11].

Additionally, analysis of commercial pet food labels reveals inconsistencies in guaranteed nutrient levels, and regulatory organizations such as the NRC, AAFCO, and FEDIAF have yet to establish consensus guidelines on recommended nutritional standards for canine obesity management. While it is recognized that caloric restriction intake through reduced fat levels and increased fiber content supports weight loss, the precise minimum and maximum macronutrient concentrations for weight-loss diets remain undefined [11,12].

Clear, evidence-based recommendations are crucial to ensure effective treatment outcomes. Therefore, this meta-analysis aims to establish minimum and maximum inclusion levels of key macronutrients—energy, protein, ether extract, total dietary fiber, and non-nitrogenous extract—to identify the nutrient ranges most effective for promoting weight loss and for optimizing body composition (lean and fat mass) in dogs on hypocaloric diets.

## 2. Materials and Methods

### 2.1. Search Strategy

This meta-analysis was designed to evaluate and correlate the nutritional components of obesity-specific diets with their respective effects on weight, fat percentage, lean body mass percentage, and total weight loss in overweight and/or obese dogs. Data collection for this study was conducted from November 2022 through May 2024.

Studies included in this analysis were identified through comprehensive searches in electronic databases and reference lists of relevant articles. Primary searches were conducted in PUBMED, Scielo, and Web of Science, with Google Scholar used to capture additional studies not indexed in other databases. The search strategy was constructed using the PICO framework (Population, Intervention, Control, and Outcomes) with the following search terms:

- Population: “obesity dogs” OR “obesity dog” AND;
- Intervention: “hypocaloric diets” OR “nutritional intervention” AND;
- Control: “diet” OR “food” OR “nutrients” AND;
- Outcomes: “weight” OR “body composition”.

**Figure 1:**
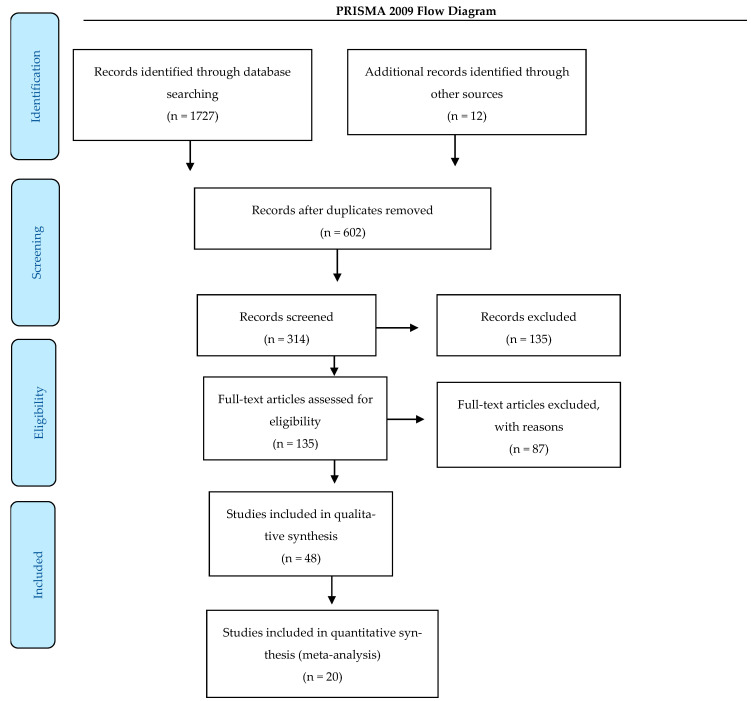
PRISMA diagram. Preferred reporting items for systematic reviews and meta-analysis (PRISMA) flow diagram identifying the total number of articles initially surveyed and the number of articles included and excluded for this systematic review [12].

The search methodology and database construction are summarized in Figure 1 and were conducted in accordance with the Preferred Reporting Items for Systematic Reviews and Meta-Analyses (PRISMA^®^) guidelines [12]. Although this meta-analysis protocol was not pre-registered, this study followed similar methodologies to those used in recently published systematic reviews and meta-analyses.

Inclusion and exclusion criteria: This meta-analysis included studies conducted on overweight and/or obese dogs, both male and female, neutered or intact, aged between 1 and 6 years. To enable linear data evaluation, we selected longitudinal studies that implemented calorie-restricted diets for weight loss in obese animals. Included studies were required to report at least the nutritional composition of the experimental diets, initial weight (pre-intervention), and final weight (post-program), along with at least one of the following variables: % lean mass, % fat mass, % total fat lost, and the mean and standard deviation of outcomes. Studies that supplemented diets with prebiotic fiber sources, probiotics, L-carnitine, antioxidants, or omegas were also considered. If studies included other variables within the same experimental trial, only the relevant data were extracted. No restrictions were applied regarding publication year.

The exclusion criteria were as follows: book references, book chapters, literature reviews, articles not available in full, studies with authors who could not be contacted, and extended abstracts, to ensure result reliability. Manuscripts involving humans, felines, or dogs with secondary obesity due to endocrine disorders or comorbidities (e.g., cancer, heart disease, renal disease) were excluded. Additionally, studies lacking details on nutritional management, guaranteed nutrient levels, or reporting means and standard deviations for statistical results were excluded.

The cutoff values for each nutrient level were established after compiling and analyzing the diet compositions in the included studies. This approach allowed for deriving intermediate nutrient levels, enabling further analyses of weight loss effects on body composition parameters.

### 2.2. Eligibility Criteria

After selecting the studies, a quality assessment was conducted, assigning scores based on the specific scientific criteria detailed below. While not all parameters were included in the scoring, relevant factors such as age, sex, breed, protocol duration, and diet composition were considered for contextual importance in the discussion. The scoring system, adapted from previous systematic reviews [13,14], rated certain criteria on a scale of 0 to 2, with 0 indicating inadequacy, 1 representing partial adequacy, and 2 indicating adequacy. Only the most pertinent parameters were scored, as shown in Table 1.

A—Sample Size: Studies with fewer than six animals per treatment group received a score of 0. Studies with 6 to 20 animals were assigned 1 point, and those with more than 20 animals were awarded 2 points.

B—Randomization: Prospective studies employing randomization were given 2 points. Studies lacking randomization or those with unclear experimental design received 1 point.

C—Sample Homogeneity: Studies were awarded 2 points if they used animals that were homogeneous in terms of breed, sex, size, and age. Studies with heterogeneous samples or those not specifying one or more of these characteristics received 1 point.

D—Analyzed Variables: Studies were awarded 2 points if they assessed initial and final weights, initial and final lean mass percentages, initial and final body fat percentages, and total weight loss. Studies analyzing only one to three of these variables were assigned a score of 1 point; and those that did not evaluate these parameters or where the results were unclear received 0 points.

E—Guaranteed Nutrient Levels: Studies that reported guaranteed levels of metabolizable energy (ME), crude protein (CP), ether extract (EE), total dietary fiber (TDF), and non-nitrogenous extracts (NFEs) in the hypocaloric diets were awarded 2 points. Studies that evaluated one to three of these variables received 1 point, while those that did not assess these parameters or provided unclear results received 0 points.

### 2.3. Data Analysis

To facilitate a structured statistical analysis, data were organized by key variables, including initial and final weight, initial and final lean mass percentage, initial and final body fat percentage, and total weight loss, in addition to nutrient levels (ME, CP, EE, TDF, NFE). Nutrient values were classified according to guaranteed nutritional levels derived from standard literature sources.

Data tabulation and statistical analyses were conducted using Microsoft Excel 365 (2019) and Review Manager 5.4.1 (2020). Results are presented as arithmetic means, standard deviations (SDs), and confidence intervals (CIs). A fixed confidence level of *p* = 0.05 was applied based on the T-test. The overall effect was analyzed using the Z-test, and treatment heterogeneity was assessed with the I^2^ test.

## 3. Results

The results were categorized based on searches across indexing databases, yielding a total of 1.727 articles (Figure 1). The review process began by removing duplicates, resulting in 314 studies for further evaluation. An additional search through the references cited in these studies identified 12 new articles. Following an abstract screening, 135 studies were excluded. The remaining 48 studies underwent full review, and 20 met the established selection criteria. The 28 excluded studies lacked results presented as means and standard deviations, which precluded their inclusion in the meta-analysis.

### 3.1. Evaluation of Data Extracted from Selected Studies

#### 3.1.1. Eligibility Criteria

After applying eligibility scoring criteria, 20 articles qualified for the meta-analysis. Additional variables, including experimental period, body condition score (BCS), breeds studied, and other factors (e.g., BCS, blood biochemical parameters, lipid profile, intake levels, hormonal profile, and gene expression) were used descriptively to support result interpretation and discussion synthesis (Table S1).

The maximum eligibility score achieved was 10 points, awarded to only one article, while the minimum score was 6, earned by six studies. All studies met the requirement of including at least six animals per treatment group. Nine studies employed randomization, and only three had homogeneous experimental groups (i.e., animals of the same sex, size, and/or age) (Table S1).

#### 3.1.2. Inclusion Criteria and Additional Information

The number of animals per treatment group ranged from 6 to 39, totaling 397 animals across all studies included in this review. Of the 20 selected articles, nine provided mean and standard deviation values for all relevant variables. However, 14 studies reported only partial data for these variables, so only data pertinent to this meta-analysis were extracted.

The number of animals per treatment group ranged from 6 to 39, resulting in a total sample size of 397 animals across all studies included in this review. Of the 20 selected articles, nine provided results as mean and standard deviation for all variables of interest. However, 14 studies did not report all the variables in this format; for these studies, only data relevant to the objectives of this meta-analysis were extracted.

### 3.2. Tables

All tables are included in the manuscript and supplementary materials: Table S1 presents eligibility scores for each article, while Tables S2 and S3 offer additional information used to enrich the discussion but not included in the statistical analysis.

Table 2 summarizes breed, sex, age, and outcomes for each study. Of the 20 studies, 11 utilized Beagle dogs, while the remaining 9 evaluated dogs of various, unspecified breeds. Additionally, 9 studies included both male and female dogs, with ages ranging from 1 to 7 years. Fourteen studies used neutered dogs, which is noteworthy as neutering is known to influence weight gain in canines.

Table S3 provides further details on the body condition assessment method, body condition score (BCS), expression of guaranteed nutrient levels, and additional dietary characteristics. No study used subjective evaluation for BCS; most employed DEXA (Dual-Energy X-ray Absorptiometry), ensuring all dogs were classified as overweight or obese. BCS scales varied (1/5 or 1/9), but this did not impact study selection or outcomes. The duration of experimental protocols ranged from 30 to 194 days. In 16 studies, nutrient levels were expressed on a dry matter basis, and inclusion levels were likewise assessed on this basis. Additionally, five studies incorporated nutraceuticals into the diets, while the remainder used conventional weight-loss diets.

Table 1 details the nutrient inclusion levels in the hypocaloric diets used in the reviewed weight-loss programs. This dataset enabled comparative analysis for the meta-analysis. Of the 20 articles, 11 reported all relevant nutritional components; the remaining articles were included, though statistical analysis was conducted only on available data. When studies employed multiple diets, each diet’s nutrient profile and outcomes were separately assessed within the meta-analysis according to the protocol used.

Data were analyzed on a dry matter basis, as 16 of the 20 articles reported their data in this format rather than in kcal. Only one article lacked caloric data for the diets. Although calorie content can be estimated, we opted not to calculate this value, given that metabolizable energy is typically measured using a bomb calorimeter, which provides precise rather than estimated values. Additionally, six articles did not report nitrogen-free extract (NFE) values, and these could not be calculated due to missing essential information, such as moisture and mineral content, required for the mathematical formula.

### 3.3. Meta-Analysis

Results are presented as weighted means and standard deviations. Most statistically significant differences were verified using the *t*-test, except for those noted with an asterisk (*) in the table.

Table 2 displays the statistical analysis results. Groups were categorized according to nutritional values and variable analyses, including weight loss, lean mass gain, and body fat reduction, measured both before initiating the weight-loss protocol and at the experiment’s conclusion. This approach enabled two comparisons within each evaluated group.

To assess the impact of nutrient inclusion percentages on weight and body composition outcomes at the end of the experimental protocol, additional stratified statistical analyses were conducted. These analyses examined calorie concentrations, as well as the percentages of protein, total digestible fiber, ether extract, and nitrogen-free extract on a dry matter basis.

**Figure 2:**
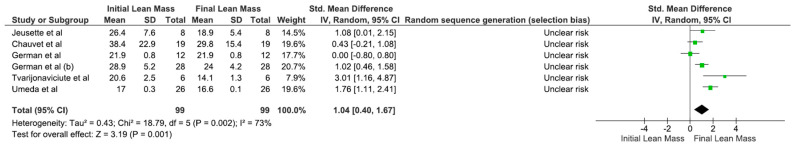
Meta-analysis of the association between the initial and final lean mass of obese dogs and metabolizable energy concentrations (<3.275 kcal). Mean: media. SD: mean, standard deviation of the mean. Total: number of animals in the sample. Weight: represented as a percentage and indicates the influence of the study on the overall result (Chauvet et al., 2011 [22]; German et al., 2007 [20]; German et al., 2010 [21]; Jeusette et al., 2005 [17]; Tvarijonaviciute et al., 2013 [24]; Umeda et al., 2006 [18]).

The most relevant results—final weight, percentage of muscle mass gain, and percentage of body fat loss—were selected for presentation. For both calorie concentration groups (less and more than 3.275 kcal), significant weight loss was observed (*p* < 0.001) (Table 2). Notably, in the <3.275 kcal group, there was a significant decrease in fat mass and a greater reduction in body fat percentage (*p* < 0.001) (Figure 2).

**Figure 3:**
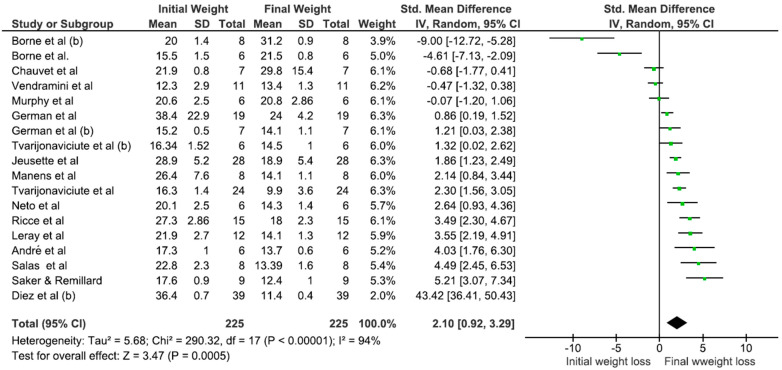
Meta-analysis of the association between the initial and final weight losses of obese dogs and crude protein concentrations (>25%). Mean: media. SD: mean, standard deviation of the mean. Total: number of animals in the sample. Weight: represented as a percentage and indicates the influence of the study on the overall result (Diez et al., 2002 [7]; Neto et al., 2018 [28]; Salas et al., 2018 [29]; Jeusette et al., 2005 [17]; Ricce et al., 2011 [23]; German et al., 2007 [20]; Chauvet et al., 2011 [22]; Leray et al., 2008 [5]; Borne et al., 1996 [15]; Murphy et al., 2020 [30]; Manens et al., 2014 [26]; Tvarijonaviciute et al., 2013 [24]; André et al., 2017 [6]; Tvarijonaviciute et al., 2012 [25]; Vendramini et al., 2020 [4]; German et al., 2010 [21]; Saker and Remillard 2005 [16]).

For crude protein levels, the total and weekly weight losses were higher in the >25% group (Figure 3), with significant heterogeneity (*p* < 0.0001), as was the reduction in body fat (Table 2). Lean mass showed a significant increase in the >25% group. However, due to limited data for crude protein levels <25% in the included studies, a direct comparison between these protein levels was not possible.

**Figure 4:**
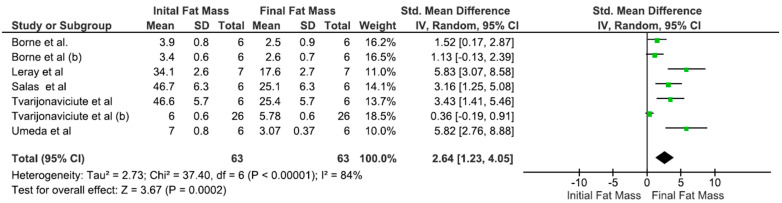
Meta-analysis of the association between the initial and final fat masses of obese dogs and total dietary fiber concentration (<12%). Mean: media. SD: mean, standard deviation of the mean. Total: number of animals in the sample. Weight: represented as a percentage and indicates the influence of the study on the overall result (Borne et al., 1996 [15]; Leray et al., 2008 [5]; Salas et al., 2018 [29]; Tvarijonaviciute et al., 2013 [24]; Tvarijonaviciute et al., 2012 [25]; Umeda et al., 2006 [18]).

Optimal results for both total weight loss (*p* < 0.0001) and body fat reduction (*p* < 0.0001) were observed with total dietary fiber contents below 12% (Figure 4). However, no significant difference in lean mass loss was found at fiber levels below 12%, in contrast to levels above 12% (Table 2).

**Figure 5:**
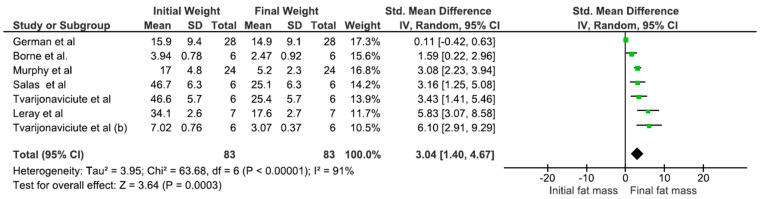
Meta-analysis of the association between the initial and final weigh loss of obese dogs and ether extract (<10%). Mean: media. SD: mean, standard deviation of the mean. Total: number of animals in the sample. Weight: represented as a percentage and indicates the influence of the study on the overall result (German et al., 2010 [21]; Borne et al., 1996 [15]; Murphy et al., 2020 [30]; Tvarijonaviciute et al., 2013 [24]; Tvarijonaviciute et al., 2012 [25]; Leray et al., 2008 [5]; Salas et al., 2018 [29]).

The analyses indicate that the group with <10% ether extract (EE) demonstrated the most substantial weight loss at the end of the experiment, as well as the highest total weight reduction (*p* < 0.001) (Table 2). Furthermore, this group exhibited a greater increase in lean mass and a highly significant reduction in body fat (Figure 5) compared to the group with EE levels exceeding 10%.

**Figure 6:**
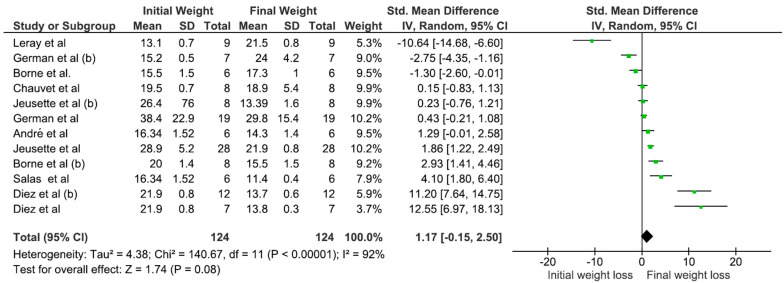
Meta-analysis of the association between the initial and final weight losses of obese dogs and nitrogen-free extract concentrations (<40%). Mean: media. SD: mean, standard deviation of the mean. Total: number of animals in the sample. Weight: represented as a percentage and indicates the influence of the study on the overall result (Leray et al., 2008 [5]; German et al., 2007 [20]; Borne et al., 1996 [15]; Chauvet et al., 2011 [22]; Jeusette et al., 2005 [17]; German et al., 2010 [21]; André et al., 2017 [6]; Jeusette et al. 2005 [17] (b); Salas et al., 2018 [29]; Diez et al., 2002 [7]).

Regarding carbohydrate concentrations in the diets, the group with non-fiber carbohydrate (NFE) levels below 40% exhibited greater weight loss (Figure 6) and a more substantial reduction in body fat, with no statistically significant loss of lean mass (Table 2). In contrast, NFE levels exceeding 40% were associated with increased weight gain, a lower percentage of weight loss, and no statistically significant reduction in body fat (Table 2).

## 4. Discussion

The composition of commercial weight-loss diets for dogs, as used across studies, is highly variable (Table 1), encompassing a wide range of caloric contents and differing levels of protein, fat, and fiber—nutrients critical for effective weight reduction. Currently, no standardized guidelines exist for these nutrient levels; however, recommendations from the European Pet Food Industry (2024) suggest that weight-loss diets should be low in calories and fat, while including high protein and dietary fiber content. In line with these guidelines, the present meta-analysis offers nutritional recommendations, estimating optimal minimum and maximum levels of macronutrients to support weight loss in obese dogs.

The studies included in this analysis varied in protocol duration, ranging from 30 to 194 days (Table S2). While protocol duration was not an exclusion criterion, we recognize this as a limitation, as extended durations typically result in more substantial weight loss. This factor should be considered in future analyses to account for its potential impact on weight-loss outcomes.

An additional limitation of this meta-analysis relates to the composition of diets used in the experimental protocols. Among the 21 articles included, five (Table S2) featured nutraceutical compounds (such as L-carnitine, omega-3, and prebiotics), which may provide supplementary benefits by enhancing weight loss or promoting lean mass gain. Similar effects may arise from individual characteristics such as age, sex, reproductive status, and breed (Table S3). Younger animals, intact males, and those with higher metabolic rates can significantly influence weight and body composition, representing another limitation of this study. To account for these variables, we incorporated them into the statistical analysis for both evaluated groups, ensuring that any potential effects on the measured variables were consistently reflected across groups.

We recognize that weight-loss diets are generally not tailored to meet the specific nutritional needs of different dog sizes but rather address obesity as a condition. Our findings aim to align with industry practices to support the enhancement of nutritional formulations and optimize outcomes for canine weight management.

The primary objective of low-calorie diets in canine obesity management is to reduce body weight while preserving lean mass and improving body composition, ensuring that the animal consumes the optimal quantities to meet all nutrient requirements. In this study, various parameters, including body weight, lean mass, body fat, and both total and weekly weight loss, were evaluated in relation to specific nutritional thresholds (e.g., < or >3.275 kcal, < or >25% CP, < or >12% NDF, < or >8.5% EE, and < or >40% NFE). Accordingly, this discussion is organized into segments that assess the effects of each nutrient level on these individual variables.

The results indicate notable changes in body characteristics and weight reduction in dogs following a weight-loss protocol. Multiple factors beyond caloric restriction can influence weight loss, such as sex, age, reproductive status (intact or neutered), as well as cultural and geographical factors [31]. For instance, younger animals, males, and intact individuals generally exhibit higher metabolic rates, which can directly impact weight and body composition, representing a limitation of this study. However, to mitigate this effect, we included these variables in the statistical analyses across both groups, ensuring that any influence on the measured parameters was evenly distributed.

It is also important to consider that the duration of the therapeutic protocol directly affects the progression and efficacy of weight loss. According to the literature [6], effective weight reduction and muscle mass preservation require a gradual, prolonged approach. Among the studies included, protocol duration varied significantly, ranging from 30 to 224 days (Table S3). Weekly weight-loss rates differed across groups, with the most significant changes observed in diets with protein levels above 25% and dietary fiber levels below 12% (Table 2). However, the groups categorized by metabolizable energy (ME) did not show significant differences, possibly due to some protocols being implemented over a shorter period (as brief as 4 weeks), which is insufficient for achieving optimal body condition and weight targets.

The ideal duration of a weight-loss program may vary based on the degree of caloric restriction and numerous additional factors, such as individual characteristics, owner compliance, diet quality, and presence of comorbidities. The literature does not specify a precise timeframe for effective results; however, it suggests that dogs with higher body condition scores (9/9) benefit from an extended period in a weight-reduction program to achieve optimal outcomes [32].

Energy concentrations below 3.275 kcal present a noteworthy outcome for consideration in canine weight-loss diets. While total weight loss in this group was lower than in the >3.275 kcal group, this effect was accompanied by a more substantial reduction in body fat and a significant increase in lean mass, clarifying the reduced overall weight-loss rate. In weight-loss protocols, it is crucial for weight reduction to be substantial while lean mass is preserved. These findings may be associated with the diet’s overall composition and relatively higher protein levels [18,33].

The data suggest that while lean mass remained comparable between groups, levels exceeding 3.275 kcal did not promote additional lean mass gain. According to the literature, an optimal weight-loss process in animals ideally leads to an increase in lean mass and a reduction in body fat [25,32]. Thus, caloric levels above 3.275 kcal may not support this benefit as effectively as levels below this threshold.

Moreover, lean muscle tissue contributes to sustaining energy expenditure, prevents the recurrence of obesity, and supports weight maintenance. Here, lean mass gain was observed at dietary protein concentrations above 25% CP (Table 2), though comparisons with protein levels below 25% were not feasible due to insufficient data for meta-analysis. These results align with findings reported in previous studies [5,6,32], which indicate that protein levels in calorie-restricted diets must meet the animal’s metabolic needs for cellular renewal. This facilitates lean mass preservation and promotes fat loss. Additionally, maintaining muscle mass during weight reduction is contingent not only on the amount of protein but also on the quality and specific amino acid profile present in the diet [33].

Significant findings on protein concentrations include higher weekly and end-of-experiment weight loss and marked reductions in body fat at levels exceeding 25% CP. These effects are likely related to the metabolic impacts of high protein inclusion in weight-loss diets. Protein appears to exert a notable thermic effect on postprandial metabolism, leading to increased caloric expenditure and a negative energy balance. Additionally, protein contributes positively to satiety in animals, which can support adherence to a calorie-restricted diet [32].

When examining body fat reduction throughout the weight-loss program, varied outcomes were observed across protein concentrations. For CP levels above 25%, the reduction in body fat percentage was significant (Table 2). Most studies suggest that protein levels exceeding 25% CP enhance weight-loss outcomes and support lean mass retention, as previously noted, although the precise metabolic mechanisms driving these changes remain to be fully understood [34].

It should be noted, however, that only two studies [17,18] evaluated protein concentrations below 25%, which may partially account for the lack of a statistically significant difference in body fat reduction between initial and final averages in this category. This limitation underscores the need for additional studies to clarify the relationship between lower protein concentrations and body composition outcomes during weight loss in dogs.

Dietary fiber content is a crucial factor in achieving favorable outcomes during a weight-loss protocol. Analysis of different fiber levels revealed that the group with <12% total dietary fiber exhibited greater total and weekly weight losses, as well as a more pronounced reduction in body fat, compared with the >12% fiber group. While it would be useful to examine the specific proportions of soluble and insoluble fibers in these diets, such data were not provided in the studies. Nonetheless, dietary fiber likely supports weight loss by diluting the caloric density of food. Additionally, the inclusion of prebiotic fibers is known to beneficially modify the gut microbiota, increasing populations of beneficial bacteria, such as Lactobacillus, Saccharomyces, and Bifidobacterium, which contribute to reduced fat absorption [28,33,35]. Despite these advantages, it is essential to manage fiber levels carefully, as excessive fiber intake can harm intestinal health, potentially causing constipation, diarrhea, or damage to the intestinal mucosa [28]. Thus, establishing maximum inclusion levels for fiber is critical to balance its benefits and risks.

Regarding nitrogen-free extract (NFE) levels, results indicated that levels exceeding 40% are associated with weight gain from baseline and do not significantly influence body fat reduction (Table 2). The role of simple sugars and complex carbohydrates has gained prominence in canine nutrition, paralleling research in human dietary studies. Similarly to findings in humans, the type and number of sugars and carbohydrates consumed by dogs can impact postprandial glycemic responses and insulin secretion [34]. Certain studies suggest that moderating starch levels may aid in glycemic control and support weight management in dogs [32,33].

Overall, few studies in the literature have explored the relationship between dietary starch levels and weight gain in dogs. It is generally understood that obesity arises from a positive energy balance, with dietary calories are primarily derived from fats. The findings of this study may therefore offer a new perspective on the nutritional management of canine obesity.

The most favorable results for weight loss, lean mass gain, and body fat reduction were observed in groups with ether extract levels below 10%. These findings highlight that fat, as the most calorie-dense macronutrient, plays a pivotal role in weight management, where restricting dietary fat is essential for achieving substantial weight loss. Diets high in lipids tend to promote greater body weight retention, even when caloric intake is reduced. Conversely, fat restriction results in lower storage of fat and triglycerides in adipose tissue, which decreases body fat percentage, facilitating weight loss and overall fat reduction [35].

In dogs, an elevated body fat percentage has been shown to inversely affect life expectancy. Maintaining an optimal body condition may extend a dog’s lifespan by approximately 2.5 years, underscoring the health benefits of effective weight management [3].

### Nutritional Recommendations

The findings of this study highlight that the most effective nutritional strategies for managing obesity in dogs involve specific dietary formulations characterized by metabolizable energy levels below 3.275 kcal/g, crude protein concentrations exceeding 25% of dry matter (DM), total dietary fiber content below 12% DM, ether extract levels under 10% DM, and nitrogen-free extract restricted to less than 40% DM (Table 3). These nutrient profiles synergistically promote significant weight loss, preservation of lean body mass, and reduction in body fat. High protein levels play a pivotal role by stimulating thermogenesis, supporting lean mass retention, and enhancing satiety. Moderate fiber content contributes to gastrointestinal health and reduces caloric density, while the limitation of fat and carbohydrates, the primary energy contributors in canine diets, is critical for achieving a negative energy balance and optimizing body composition. These results underscore the importance of precise macronutrient formulations in therapeutic diets to achieve effective weight loss while maintaining overall health and extending the longevity of obese dogs.

## 5. Conclusions

The findings of this meta-analysis demonstrate that optimal nutrient composition can guide the development of industrial strategies for formulating diets to achieve effective weight loss in obese dogs. Despite variability among studies, this analysis established minimum and maximum inclusion levels for nutrients in hypocaloric diets. Differences in body composition were observed across all stratified groups, with findings applicable across sexes, ages, and breeds. Specifically, a caloric limit of 3.275 kcal, ether extract capped at 10%, and non-nitrogenous extracts limited to 40%, combined with a minimum of 25% protein and total dietary fiber levels above 12%, positively influenced weight loss and body composition, enhancing lean mass preservation and reducing body fat. These adjustments contributed to weight reduction while minimizing adverse effects and improving the quality of life for obese dogs. Other nutrient levels did not show significant effects for weight management. This precise nutritional approach advances the fight against canine obesity by promoting a formula that not only supports body fat reduction but also sustains the overall health and vitality of dogs.

## Figures and Tables

**Table 1:** Diet composition used in the studies. The values are presented in metabolizable energy (kcal/kg), crude protein (CP%), ether extract (EE%), total dietary fiber (TDF), and nitrogen-free extract (NFE).

Author	ME (kcal/kg)	CP%	EE%	TDF%	NFE%
Vendramini et al. (2020) [4]	2.979	36.9	10.27	10.37	NP
Leray et al. (2008) [5]	3.081	21.0	5.0	5.0	55.0
André et al. (2017) [6]	2.930	37.5	11.4	13.8	13.4
Diez et al. (2002) [7]	2.770 2.340	47.5 23.8	NP NP	30.8 38.6	5.3 23.9
Borne et al. (1996) [15]	NP NP	25.6 37.5	7.0 17.9	26.5 2.9	35.9 35.6
Saker and Remillard (2005) [16]	2.780	22.7	5.9	15.1	48,5
Jeusette et al. (2005) [17]	3.090	32.0	6.7	10.7	43.3
Umeda et al. (2006) [18]	2.866 4.132	34.0 24.0	9.5 16.1	27.0 NP	14.8 38.0
Yoo et al. (2006) [19]	3.275	37.1	10.9	12.2	21.0
German et al. (2007) [20]	3.275	37.1	10.9	21.6	21.0
German et al. (2010) [21]	3.272	34.0	10	19.8	19.3
Chauvet et al. (2011) [22]	3.275	37.1	10.9	12.2	21.0
Riicce et al. (2011) [23]	3.526	37.4	11.0	3.0	38.8
Tvarijonaviciute et al. (2013) [24]	2.886	34.0	9.5	3.0	NP
Tvarijonaviciute et al. (2012) [25]	3.275	34.0	10.0	8.2	NP
Manens et al. (2014) [26]	3.011	34.6	9.0	13.1	NP
Vitger et al. (2016) [27]	NP	30.0	9.5	16.5	NP
Neto et al. (2018) [28]	2.796	32.0	9.0	22.0	NP
Salas et al. (2018) [29]	2.870	33.7	8.3	10.6	20.8
Murphy et al. (2020) [30]	733.0	25.5	10.0	NP	NP

CP: crude protein; EE: ether extract; NFE: nitrogen-free extract; ME: metabolizable energy; NP: not provided; TDF: total dietary fiber.

**Table 2:** Effects of different nutritional inclusion levels on weight variables and body characteristics (lean mass, fat mass) of obese dogs.

Nutrient	ME (<3.275) kcal/Day			ME (>3.275) kcal/Day		
Effect	n	Mean ± SD	SD^2^	n	Mean ± SD	SD^2^
Start body weight (kg)	205	24.31 ± 2.94 *	2.87	99	26.04 ± 114.13 *	83.60
End body weight (kg)	205	20.11 ± 2.80 *		99	21.9 ± 53.21 *	
Weekly weight loss	205	0.3	0.21	99	0.3	0.25
*p* value	<0.001			<0.001		
Start lean mass (%)	47	10.66 ± 3.53 *	8.27	47	16.63 ± 86.67	84.12
End lean mass (%)	47	16.61 ± 13.05 *		47	15.63 ± 81.37	
*p* value	<0.001			0.3		
Start fat mass (%)	67	22.98 ± 9.92 *	6.85	79	14.57 ± 82.72 *	59.82
End fat mass (%)	67	12.19 ± 3.79 *		79	9.51 ± 36.93 *	
*p* value	<0.001			<0.001		
Total weight loss (%)	89	7.37 ± 2.12 *	8.36	87	15.51 ± 14.77 *	8.36
*p* value	<0.001					
**Nutrient**	**CP (<25%)**			**CP (>25%)**		
**Effect**	**n**	**Mean ± SD**	**SD^2^**	**n**	**Mean ± SD**	**SD^2^**
Start body weight (kg)	77	24.69 ± 1.97 *	1.57	297	24.53 ± 63.56 *	49.39
End body weight (kg)	77	22.71 ± 1.17 *		297	19.76 ± 35.22 *	
Weekly weight loss	77	0.20	0.26	297	0.43	0.17
*p* value	<0.001			<0.001		
Start lean mass (%)	NE	NE	NE	106	13.67 ± 41.58 *	42.39
End lean mass (%)	NE	NE		106	15.48 ± 43.21 *	
*p* value	NE			<0.001		
Start fat mass (%)	36	4.74 ± 2.65 *	1.84 *	144	28.90 ± 55.56	43.90
End fat mass (%)	36	5.01 ± 1.04 *		144	20.87 ± 32.26	
*p* value	<0.001			0.2		
Total weight loss (%)	72	4.12 ± 0.03 *	22.96	196	15.70 ± 31.32 *	22.96
*p* value	<0.001			<0.001		
**Nutrient**	**FDT (<12%)**			**TDF (>12%)**		
**Effect**	**n**	**Mean ± SD**	**SD^2^**	**n**	**Mean ± SD**	**SD^2^**
Start body weight (kg)	140	28.85 ± 1.15 *	1.91	182	25.75 ± 98.20 *	76.51
End body weight (kg)	140	20.07 ± 2.758 *		182	21.49 ± 58.83 *	
Weekly weight loss	140	0.81	0.53	182	0.23	0.23
*p* value	<0.001			<0.001		
Start lean mass (%)	25	10.29 ± 0.85	0.79	47	16.63 ± 86.87	84.12
End lean mass (%)	25	10.0 ± 0.73		47	15.63 ± 81.37	
*p* value	0.01			0.3		
Start fat mass (%)	63	16.51 ± 7.47 *	7.49	83	28.28 ± 84.95 *	63.59
End fat mass (%)	63	9.93 ± 7.52 *		83	20.07 ± 42.23 *	
*p* value	<0.001			<0.001		
Total weight loss (%)	75	9.99 ± 37.11 *	25.77	134	15.44 ± 19.48 *	25.77
*p* value	<0.001			<0.001		
**Nutrient**	**NFE (<40%)**			**NFE (>40%)**		
**Effect**	**n**	**Mean ± SD**	**SD^2^**	**N**	**Mean ± SD**	**SD^2^**
Start body weight (kg)	160	23.03 ± 10.50 *	80.47	36	18.11 ± 3.50 *	3.46
End body weight (kg)	160	19.23 ± 55.45 *		36	20.77 ± 3.43 *	
Weekly weight loss	160	0.43	0.26	36	0.05	0.008
*p* value	<0.001			<0.001		
Start lean mass (%)	72	14.89 ± 59.49	57.63	NE	NE	NE
End lean mass (%)	72	13.79 ± 55.77		NE	NE	
*p* value	0.2			NE		
Start fat mass (%)	72	155.7 ± 91.59 *	65.58	36	5.19 ± 0.86	0.94
End fat mass (%)	72	8.84 ± 39.57 *		36	5.01 ± 1.04	
*p* value	<0.001			0.21		
Total weight loss (%)	124	18.77 ± 44.30 *	34.48	36	1.64 ± 0.01 *	34.48
*p* value	<0.001			<0.001		
**Nutrient**	**EE (<10.0%)**			**EE (>10.0%)**		
**Effect**	**n**	**Mean ± SD**	**SD^2^**	**n**	**Mean ± SD**	**SD^2^**
Start body weight (kg)	180	26.39 ± 6.62 *	6.06	240	18.70±9.52 *	7.94
End body weight (kg)	180	22.61 ± 5.40 *		240	16.77±5.47 *	
Weekly weight loss	180	0.48	1.05	240	0.83	1.61
*p* value	<0.001			<0.001		
Start lean mass (%)	71	10.63 ± 37.69 *	39.70	25	15.97 ± 66.59	66.51
End lean mass (%)	71	14.08 ± 41.73 *		25	15.84 ± 66.54	
*p* value	<0.001			0.5		
Start fat mass (%)	42	34.05 ± 23.52 *	18.18	164	18.70 ± 9.52 *	7.94
End fat mass (%)	42	10.62 ± 12.72 *		164	16.77 ± 5.47 *	
*p* value	0.11			<0.001		
Total weight loss (%)	42	19.40 ± 14.53 *	24.62	200	11.17 ± 42.75 *	24.62
*p* value	<0.001			<0.001		

* There was statistical difference in the results by the *t* test; n: sample size; SD: standard deviation; SD^2^: mean standard deviation difference; CP: crude protein; EE: ether extract; NFE: nitrogen-free extract; ME: metabolizable energy, NE: not evaluated; TDF: total dietary fiber.

**Table 3:** Nutritional recommendations for the treatment of obese dogs.

ME (kcal)	CP%	EE%	TDF%	NFE%
3.275	25	10	12	40

CP: crude protein; EE: ether extract; NFE: nitrogen-free extract; ME: metabolizable energy; TDF: total dietary fiber.

## Data Availability

Not applicable.

## Supplementary Materials

The following supporting information can be downloaded at https://www.mdpi.com/article/10.3390/ani15020210/s1.
