# Supplementary material for: Impact of Hypocaloric Diets on Weight Loss and Body Composition in Obese Dogs: A Meta-Analysis
Source: Animals (Basel). 2025 Jan 14;15(2):210. doi: 10.3390/ani15020210 (PMC11759159; doi:10.3390/ani15020210)
Supplement: Supplementary file 1 [file animals-15-00210-s001.zip › animals-3296473-supplementary.pdf]

Systematic Review and Meta-analysis

Supplementary Materials

Impact of Hypocaloric Diets on Weight Loss and Body Composition in Obese Dogs: A Meta-Analysis

Vanelli Karoline [1*], Winsneski, Rafael Fernando[2]; Estevão Camila[3] Mayer, Fernanda Caroline[4]; Costa, Leandro, Batista[5]; Webber, Saulo Henrique[6]; Pimpão, Cláudia, Turra[7*]

PRISMA 2009 Flow Diagram

Table S1.

Author	A	B	C	D	E	Total
André et al. (2017) [6]	1	2	1	1	2	7
Borne et al. (1996) [16]	1	1	1	2	2	7
Chauvet et al. (20110 [24]	1	1	1	1	2	6
Diez et al. (2002) [7]	1	1	1	1	2	6
German et al. (2007) [21]	1	2	1	2	2	8
German et al. (2009) [23]	2	1	1	2	2	8
Jeusette et al. (2005) [18]	1	1	1	1	2	6
Leray et al. (2008) [5]	1	1	2	2	2	8
Manens et al. (2013) [27]	1	2	1	1	2	7
Murphy et al. (2020) [32]	2	2	2	2	2	10
Neto et al. (2018) [30]	1	2	1	1	2	7
Riicce et al. (2011) [25]	1	2	1	1	2	7
Saker and Remillard (2005) [17]	2	2	1	1	2	8
Salas et al. (2018) [31]	1	2	1	2	2	8
Tvarijonaviciute et al. (2012)a [26]	1	1	1	1	2	6

**Tvarijonaviciute et al. (2012)b [27]**	1	1	1	1	2	6
**Umeda et al. (2006) [19]**	2	1	1	1	2	7
**Vendramini et al. (2020) [2]**	1	2	1	2	2	8
**Vitger et al. (2016) [29]**	1	1	1	1	2	6
**Yoo et al. (2006) [20]**	1	1	2	2	2	8

Table S2. Breed information, sex, age, reproductive condition of the dogs, and the outcomes studied from the selected articles.

**Author**	**Breed**	**Sex**	**Age**	**Reproductive Condition**	**Outcomes**
**André et al. (2017) [6]**	Beagle s	M/F	3,2	Neutered	Body weight; body composition; BCS; postprandial glycaemic and insulinaemic kinetics; in vitro RAG of diets
**Borne et al. (1996) [16]**	NP	M	Young adult	Neutered	Body weight, body composition; serum lipid, insulin, and plasma glucose Blood pressure
**Chauvet et al. 2011 [24]**	Mixed breeds	M/F	6,0	Neutered	Body weight; BCS; exercise and lifestyle changes
**Diez et al. (2002) [7]**	Beagles	M/F	6,0 6,0	Neutered and intact	Body weight; body composition; BCS
**German et al. (2007) [21]**	Mixed breeds	M/F	6,5	NP	Body weight; body composition; weight program factors.
**German et al. (2009) [23]**	Mixed breeds	M/F	5-6,5	Intact and neutered	Body weight; BCS; body composition
**Jeusette et al. (2005) [18]**	Beagles	M/F	1-9	Neutered and intact	Body weight; BCS; glucose, insulin, cholesterol, triglyceride, lipoproteins, ghrelin, leptin nonesterified fatty acid; energy consumption
**Leray et al. (2008) [25]**	Beagles	F	Adult	Neutered	Body weight; body composition, euglycaemic-hyperinsulinaemic; biopsies, RNA extraction; PCR
**Manens et al. (2013) [28]**	Beagles	NP	Adult	NP	Body weight; arterial blood gas; six-minute walk test
**Murphy et al. (2020) [32]**	Beagles	M	2,0	Neutered	Body weight; BCS; body composition; caloric consumption; treatment with leucine, isoleucine; valine and pyridoxine nutraceutical
**Neto et al. (2018) [30]**	Mixed breeds	M/F	5,5	NP	Body weight; BCS; arterial blood gases; respiratory evaluation
**Riicce et al. (2011) [25]**	Beagles	M/F	3-9	Intact and neutered	Body weight; BCS; lipidic plasma profile; glucose and insulin concentration
**Saker and Remillard (2005) [17]**	NP	M/F	2-12	Neutered	Body weight; BCS; computerized obesity management program efficacy
**Salas et al. (2018) [31]**	Beagles	M/F	10	Neutered	Body weight; BCS; body composition; rRNA fecal; blood and serum biochemistry; phylogenetic and functional analysis

**Tvarijonaviciute et al. (2012)a [26]**	Beagles	F	5,2-6,5	Intact	Body weight; BCS; body composition; two-dimensional polyacryllamide gel electrophoresis; two-dimensional electrophoresis gel image analysis; mass spectrometry identification; mass spectrometry data analysis
**Tvarijonaviciute et al. (2012)b [27]**	Beagles	F	3,2-6,1	Intact	Body weight, BCS; serum total cholesterol, HDL, LDL, TG; fructosamine; IGF-1; glucose; insulin; insulin:glucose; adiponectin; haptglobolin; CRP; ceruloplasmin; IgG; IgM; IgA
**Umeda et al. (2006) [19]**	Beagles	M/F	4,9	Intact	Body weight; dietary intake; digestibility; changes in body fat mass, serum lipid concentration; diacylglycerol (DAG) as a potentially effective ingredient for canine weight control without caloric restriction
**Vendramini et al. (2020) [4]**	Mixed breeds	F	4,7	Neutered	Body weight; body composition; complete blood; biochemical profile, gene expression (resistiin, adiponectin, leptin); inflammatory profile (TNF-α, IL-6, IL-8, IL-10)
**Vitger et al. (2016) [29]**	Medium and large breeds	M/F	7,6	Neutered	Body weight; body composition; BCS; glucose, plasma insulin, and leptina, IL-2, IL-6, IL-8, MCP-1; hematology and biochemistry profile
**Yoo et al. (2006) [20]**	Labrador Retrievers	M	2,2	Intact	Body weight; body composition; energy intake; DEXA; energy expenditure

BCS: body condition score; F: female; M: male; NP: not provided.

Table S3. Causes of obesity, method of body assessment, diet characteristics, and the physical activity protocol were measured in the 20 studies included in the meta-analysis.

Author	Body Compositio n	BCS	Weight Loss Period (days)	Nutritional Guarantee Levels	Diet
**André et al. (2017) [6]**	Deuterium	Overweight Obesity	98	DM	HP; MC
**Borne et al. (1996) [16]**	DEXA	Obesity	63<br>63	DM	HF; LFB<br>LF; HFB
**Chauvet et al. 2011 [24]**	NP	5/5	86	DM/kcal	CD
**Diez et al. (2002) [7]**	Deuterium	4/5	164	DM	HP; LS<br>CD
**German et al. (2007) [21]**	DEXA	Obesity	144	DM/kcal	CD with l-carntine
**German et al. (2009) [23]**	DEXA	Obesity	NP	DM/kcal	CD with l-carnitine
**Jeusette et al. (2005) [18]**	NP	Obesity	30	DM	HP; LE

**Leray et al. (2008) [25]**	Deuterium	Obesity	78	DM	CD
**Manens et al. (2013) [28]**	NP	Obesity	121	DM	LE
**Murphy et al. (2020) [32]**	DEXA	Obesity	91	DM	CD
**Neto et al. (2018) [30]**	NP	8/9	112	DM	CD
**Riicce et al. (2011) [25]**	NP	8/9	182	DM	CD with sc-FOS
**Saker and Remillard (2005) [17]**	NP<br>NP	7/9	90<br>180	Kcal	CD
**Salas et al. (2018) [31]**	DEXA	9/9	119	DM	LF; HP; LS; HFB
**Tvarijonaviciute et al. (2012)a [26]**	Deuterium	Obesity	91	DM	CD
**Tvarijonaviciute et al. (2012)b [27]**	Deuterium	Obesity	91	DM	CD
**Umeda et al. (2006) [19]**	Deuterium	4/5	56	DM	CD with DAG
**Vendramini et al. (2020) [4]**	Deuterium	9/9	194	DM/kcal	CD
**Vitger et al. (2016) [29]**	DEXA	6/9	84	DM	CD
**Yoo et al. (2006) [20]**	DEXA	Obesity	60	DM	LF; HF

Matter; HF: high fat; LF: low fat; HFB: high fiber; LFB: low fiber; HP: high protein; LS: low starch; CD: commercial diet; HE: high energy; MFB: medium fiber; sc-FOS: short-chain fructooligosaccharides; RD: reduced calories; MC: medium carbohydrates; NP: not provided; LE: low energy.
